# Regulation of stem cell fate and function by using bioactive materials with nanoarchitectonics for regenerative medicine

**DOI:** 10.1080/14686996.2022.2082260

**Published:** 2022-06-22

**Authors:** Wei Hu, Jiaming Shi, Wenyan Lv, Xiaofang Jia, Katsuhiko Ariga

**Affiliations:** a School of Pharmaceutical Sciences (Shenzhen), Shenzhen Campus of Sun Yat-sen University, Shenzhen P. R. China; b International Center for Materials Nanoarchitectonics (MANA), National Institute for Materials Science (NIMS), Ibaraki, Japan; c Department of Advanced Materials Science, Graduate School of Frontier Sciences, the University of Tokyo, Kashiwa Japan

**Keywords:** Stem cell, differentiation, immunoregulation, organoid, nanoarchitectonics

## Abstract

Nanoarchitectonics has emerged as a post-nanotechnology concept. As one of the applications of nanoarchitectonics, this review paper discusses the control of stem cell fate and function as an important issue. For hybrid nanoarchitectonics involving living cells, it is crucial to understand how biomaterials and their nanoarchitected structures regulate behaviours and fates of stem cells. In this review, biomaterials for the regulation of stem cell fate are firstly discussed. Besides multipotent differentiation, immunomodulation is an important biological function of mesenchymal stem cells (MSCs). MSCs can modulate immune cells to treat multiple immune- and inflammation-mediated diseases. The following sections summarize the recent advances of the regulation of the immunomodulatory functions of MSCs by biophysical signals. In the third part, we discussed how biomaterials direct the self-organization of pluripotent stem cells for organoid. Bioactive materials are constructed which mimic the biophysical cues of *in vivo* microenvironment such as elasticity, viscoelasticity, biodegradation, fluidity, topography, cell geometry, and etc. Stem cells interpret these biophysical cues by different cytoskeletal forces. The different cytoskeletal forces lead to substantial transcription and protein expression, which affect stem cell fate and function. Regulations of stem cells could not be utilized only for tissue repair and regenerative medicine but also potentially for production of advanced materials systems. Materials nanoarchitectonics with integration of stem cells and related biological substances would have high impacts in science and technology of advanced materials.

## Graphical Abstract

**Figure:**
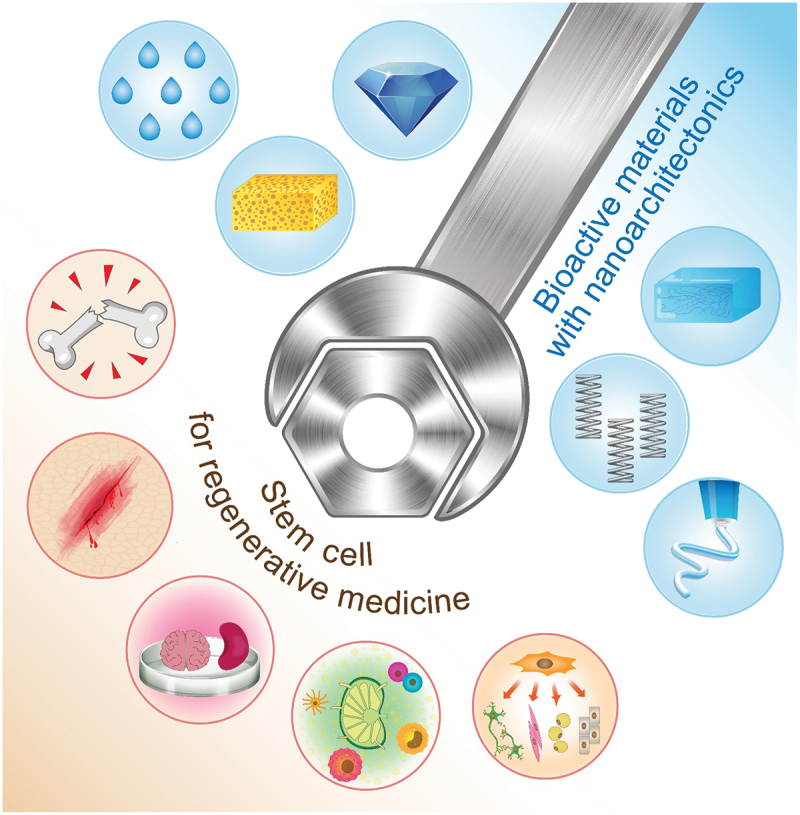

## 1 Introduction

In order to develop functional materials, it is important to prepare the materials through fundamental organic chemistry [1–3], inorganic chemistry [4–6], polymer chemistry [7–9], supramolecular chemistry [10–12], material science [13–16], and biologically related science [17–19]. Besides the materials themselves, the internal structure of the materials and the organized mode of the components have a significant impact on the efficiency and selectivity of their functions. The important effect of nanostructure on the functions of materials has received more attention with the development of nanotechnology. The development of nanotechnology has revealed the detailed structure of atoms, molecules, and nanomaterials [20,21], as well as their physical properties [22,23] and their dynamic behaviours [24,25]. The understanding of the properties of nanostructures has provided the basis for assembling functional materials. It should be possible to rationally assemble structures from atomic, molecular, and nanomaterial units to create ideal functional structures. Attempts to assemble molecules to create functional structures are being basically considered in supramolecular chemistry and other related fields [26,27]. However, it is necessary to create a new paradigm that is not limited to molecular recognition and self-assembly, but rather to a more comprehensive approach to creating functional materials from nanomaterials. This will be created by integrating nanotechnology with other research fields, such as organic chemistry, materials science, supramolecular chemistry, microfabrication technology, and bio-related technology. In this role, nanoarchitectonics has emerged as a post-nanotechnology concept [28,29], proposed by Masakazu Aono [30,31], just as Richard Feynman proposed nanotechnology [32,33]. As one of the applications of nanoarchitectonics, this review paper discusses the control of stem cell fate and function as an important issue. This could also pave a way to nanoarchitectonics for hybridization of living matters and non-living materials.

**Figure 1.**
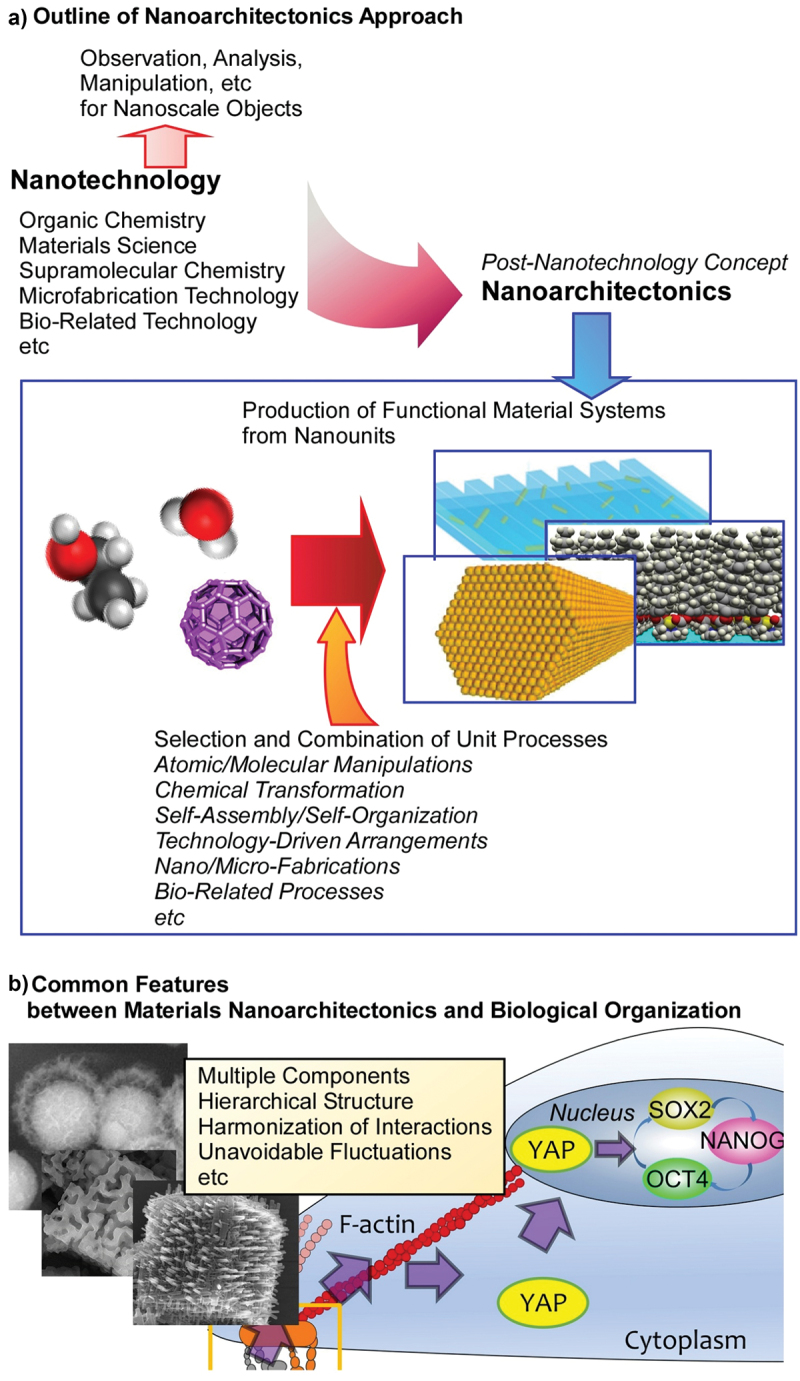
Nanoarchitectonics strategy and its similarity to biological system: (A) outline of nanoarchitectonics approach; (B) common features between materials nanoarchitectonics and biological organization.

In nanoarchitectonics strategies, functional materials are architected from nano-units such as atoms, molecules, and nanomaterials though selection and combination of unit processes including atomic/molecular manipulations, chemical transformation, self-assembly/self-organization, technology-driven arrangements, nano/micro-fabrications, and bio-related processes [34,35] (Figure 1A). Because the proposed strategy can be adaptable for a wide range of materials and functional targets, nanoarchitectonics-based approaches can be used in various research fields such as material productions [36–38], structural regulations [39–41], catalyses [42–44], sensors [45–47], devices [48–50], energy-related applications [51–53], environmental usages [54–56], biochemical investigations [57–59], and biomedical applications [60–62]. With this universal applicability, nanoarchitectonics methodology can be regarded as a method for everything in materials science [63] like a theory of everything in physics [64,65].

Probably, the ideal form of nanoarchitectonics can be seen in living systems [66]. In a biological system, a very large number of functional molecules are assembled to create a highly efficient, selective, and rational functional systems [67,68]. Although the behaviour of individual functional molecule is perturbed by thermal fluctuations, the functions produced by their interactions are highly efficient in biological systems, which are created because of hierarchical and rational organization of functional molecules. The concept of nanoarchitectonics encompasses exactly such characteristics seen in biological systems (Figure 1B). The behaviour of biomolecules as well as nanoscale molecules and materials can occur with uncertainties such as thermal fluctuations, stochastic distributions, and quantum effects. They are often uncontrollable. Therefore, the process of assembling various elements into functional structures in nanoarchitectonics integrates the individual elements so that they harmonize, rather than simply putting them together [69,70]. This mechanism in nanoarchitectonic processes is similar to the characteristics of concept formation and functional expression in biological systems. In addition, the fabrication of functional structures in nanoarchitectonics combines various processes in multiple steps, rather than just the traditional equilibrium-based self-assembly. For example, molecular self-assembly is combined with artificial thin-film formation processes such as Langmuir-Blodgett (LB) method [71–74], layer-by-layer (LbL) assembly [75–77], and microfabrication [78,79]. As a result, it has the advantage of forming hierarchical structures that cannot be obtained by simple self-assembly [80]. This is also similar to the formation of functional structures in biological systems.

The syntheses and organization of biomaterials and related materials have also been of great interest and have been widely studied [81,82]. For example, formation of helical structures and their related functions of biomolecules and/or synthetic polymer mimics have been actively researched even since the early 1950s [83]. Various examples of research suggest that biomaterials can be a component of nanoarchitectonics. Because nucleic acids, mainly DNA and RNA, can be assembled into various possible forms based on the strict base pairing rule [84,85], programmed structure formations are possible with nucleic acids as seen in DNA origami [86,87]. Well-defined functional motifs such as G-quadruplex-based functional structures [88,89] and interlocked supramolecular structures [90] have been architected. Conjugate structures such as peptide nucleic acids as artificial mimics of DNA [91] and polyimide/DNA conjugates [92] are subjected for bio-related applications. High hydrogen bonding capability of peptides is also highly useful for nanoarchitectonics for regular structures. Effects of peptide sequences in molecular assemblies in liquid crystalline phases [93] and stereochemical effect of short peptides in molecular assemblies [94] becomes subjects in basic science. Well-designed peptide sequences can be precious structural units in dynamic tubular structures [95] and supramolecular structures with metals such as polyhedral links, torus knots, and a poly[*n*]catenane [96]. Manipulation of amino acid residues in peptide designs is useful for regulation of their binding in target proteins [97]. Peptide aggregations are used for detection and imaging of Alzheimer’s disease biomarkers [98]. The other biomolecular units were also used to create functional structural units as seen in the preparation of robust gels through assembly of cello-oligosaccharide networks [99] and fabrication of nanotubes with well-controlled diameters with sugar-featured lipid molecules [100]. Proteins are used as nanoarchitectonic units for formation of various structures thermo-controllable composite structures of ferritin and poly(*N*-isopropylacrylamide) [101] and glycosylated artificial metalloenzymes [102]. Nano-confined environments formed with ferritin cages can be utilized as reactors for controlled metal cluster nanoparticles [103]. Biomaterials are conjugated with artificial structures such as nanoporous materials [104–106] and devices such as electrodes and membranes [107–109] to provide bioprocess-based sensors and reactors.

As seen in these examples, biomolecules are excellent unit materials for nanoarchitectonic procedures. However, it is difficult with current technology to create cell-like functional entities only from these biomolecules. From demands for cell-like high-level functions, there are attempts to create tissue-like organizations from living cells themselves. The methodology for live cell assembly has in fact been developed, as seen in the case of cell sheet technologies [110,111] and LbL live cell assembly [112,113]. It is conceivable to not only organize the cells themselves but also to control the cell fate and function through contacts with external substances and their organized structures. Hybridization of artificial objects and living cells paves the way to nanoarchitectonics with function relays between living substances and non-living matters. An attractive target cell for this purpose would be the stem cell. Stem cells can be differentiated into a variety of cell types with possible mechanistic inputs from external materials and structures. In these cases, information of materials and structures can be transmitted to living matters. So far, the effects of various materials and structures on stem cells have been extensively studied [114,115]. Magnetic nanoarchitectonic materials are used to guide the stem cell differentiation [116,117] and multicellular 3D spheroids [118]. Those studies will be the cornerstone of nanoarchitectonics using stem cells.

Based on these backgrounds, this review will provide an overview of stem cell regulation, particularly by various biomaterials and their nanoarchitected structures. In the sections following this introduction, (i) bio-materials for regulation of stem cell fate will be discussed from viewpoints of elasticity, viscoelasticity, degradation, fluidity, topography, cell geometry, and chemical properties. In the following sections, (ii) biomaterials for immunoregulation and (iii) biomaterials for the direction of the self-organization of pluripotent stem cells for organoid are discussed. For hybrid nanoarchitectonics involving living cells, it is very important to understand how biomaterials and their nanoarchitected structures regulate behaviours and fates of stem cells.

## 2 Biomaterials for regulation of stem cell fate

### 2.1 Elasticity

Stem cell fate is historically believed to be regulated by genetic and biochemical factors such as soluble induction factor. Currently, biophysical cues are increasingly being recognized as new factors to regulate stem cell behaviours and fate. In 2006, Engler et al. found that when cultured on collagen I coated polyacrylamide (PAAm) hydrogels of varying elasticity, the differentiation of mesenchymal stem cells (MSCs) is directed by tissue-level matrix stiffness [119]. On soft matrices like brain tissue, MSCs show neuronal differentiation; on stiffer matrices like muscle tissue, MSCs show myogenic differentiation; and on rigid matrices like bone, MSCs prove osteogenic differentiation. Mechanically, cells sense the matrix stiffness through integrin-based adhesions. Integrins form clusters, cause the conformation changes of mechanosensitive proteins like talin and vinculin, activate the mechanosensitive ion channels, and then activate the downstream transcription factor activity [120]. To construct an optimal mechanical microenvironment for culturing pluripotent stem cells, Chalut’s group modified PAAm hydrogel protocol by addition of 6-acrylamidohexanoic acid (AHA) as co-factor [121]. The carboxyl terminal groups of the AHA are covalently linked to extracellular matrix (ECM) protein through a carbodiimide reaction, and thus form stable ECM–substrate attachment. They demonstrate that these soft StemBond hydrogels provide a strong attachment but a low cytoskeletal tension to promote cell attachment, pluripotency, and self-renewal of mouse embryonic stem cells (ESCs) and human induced pluripotent stem cells (hiPSCs) in minimal media conditions (i.e. a single chemical inhibitor).

### 2.2 Viscoelasticity

Living tissue is not a perfect elastic solid, and instead shows a time-dependent mechanical viscoelastic response [122]. To mimic the viscoelastic behaviours of living tissues, Chaudhuri et al. prepared a nanoarchitecture of alginate hydrogels with varying stress relaxation properties and the same initial elastic modulus [123]. They found that faster stress relaxation enhances cell spreading and proliferation of fibroblasts. The rate of relaxation also regulates MSC differentiation. The osteogenically differentiated MSCs form an interconnected mineralized collagen-1-rich matrix like bone. The effect of stress relaxation is mediated through RGD ligand binding and clustering as well as actomyosin contractility. They further developed a hyaluronic acid–collagen interpenetrating network hydrogel through dynamic covalent hydrazone crosslinking [124]. It captures two key features of native ECM: tuneable stress relaxing and fibrillary structure. Similarly, faster relaxation in the hydrogels promotes cell spreading, fibre realignment and focal adhesion formation.

**Figure 2.**
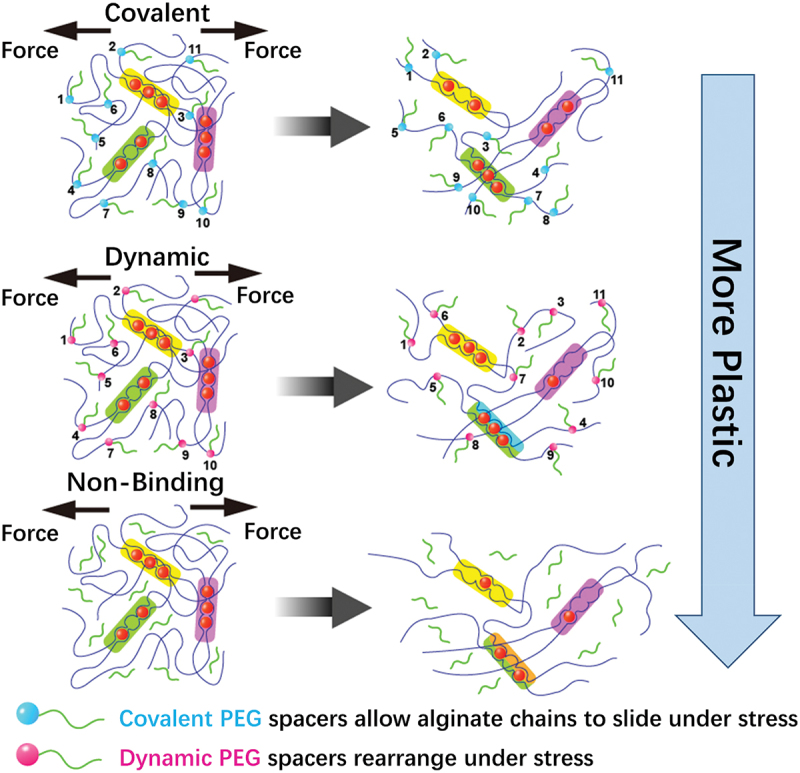
By incorporating pendant PEG to alginate that are either bound covalently, dynamically through Schiff base formation, or simply encapsulated (nonbinding), the irreversible plastic deformations can be controlled with respect to the Young’s modulus and stress relaxation.

Irreversible plastic deformation and stress relaxation are usually coupled in biomaterial system, making it difficult to discern which factor drives cell behaviour. As shown in Figure 2, Grolman et al. developed a poly(ethylene glycol) (PEG)-alginate hydrogel system to tune matrix plasticity independently of stress relaxation and modulus [125]. Moderate network plasticity enhances cell spreading to the greatest degree, while this trend is altered by inhibiting actomyosin contractility. Theoretical simulations also show strong relationship between experimental cell spreading results and ECM plasticity.

### 2.3 Degradation

For commonly used covalently crosslinked hydrogels, the dynamic mechanical properties are often derived from cell-mediated degradation. Khetan et al. encapsulated human mesenchymal stem cells (hMSCs) in covalently crosslinked hyaluronic acid hydrogels subjected to a multi-step crosslinking protocol, from cell–mediated–degradable to non-degradable [126]. They demonstrate that the differentiation of hMSCs is directed by the generation of traction force mediated through cellular degradation of matrix. Furthermore, their work emphasizes the type of hydrogel underlying the mechanism by which stem cells respond to biophysical cues. Heilshorn et al. demonstrate that the maintenance of neural progenitor cell (NPC) stemness in 3D hydrogels is dependent on matrix degradability but interestingly is independent of cytoskeletal tension and integrin-binding ligand clustering [127]. According to their work, the underlying mechanism is increased cadherin-mediated cell–cell contact and activating β-catenin signalling. Moreover, they demonstrate that NPC proliferation and differentiation also require increased degradability. Following this study, they further clarify the role of matrix remodelling on NPC differentiation and maturation [128]. Permitting 7-day matrix remodelling prior to induction of differentiation, NPCs can differentiate into astrocytes or mature functional neurons. It is attributed to up-regulating YAP expression via cadherin-mediated cell–cell contact.

### 2.4 Fluidity

**Figure 3.**
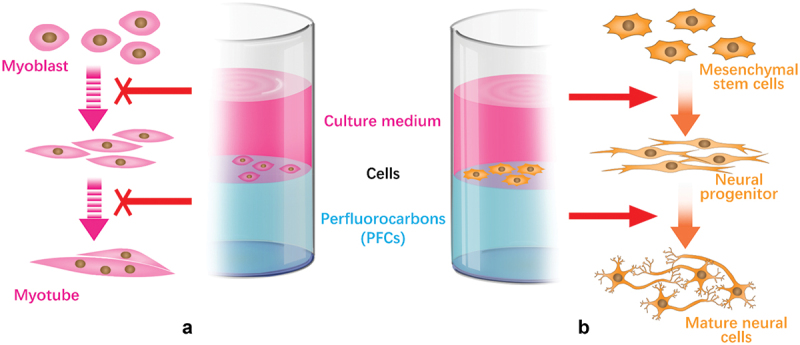
Schematic illustrations of cell culture at the liquid−liquid interfaces for control of cellular differentiation. (A) Myogenic differentiation was suppressed on fluid interface. (B) Interfacial culturing induced hMSCs differentiate into neural cells.

The interface between stem cell and ECM is dynamic. Stem cells remodel ECM and cause spatial rearrangements of the ECM, which feedback to affect stem cell behaviour and fate decision. Recently, Jia et al. developed an adaptive culturing system based on a liquid–liquid interface. They found a protein monolayer self-assembled at the liquid–liquid interface with mechanical properties strong enough to support hMSCs spreading [129]. Higher stiffness of protein nanosheets on perfluorotributylamine promotes cell spreading and adhesion in relative to that on perfluorodecalin interface. To investigate the biological effects of a fluid substrate, Minami et al. cultured C2C12 cells at a water–perfluorocarbon (PFC) interface (Figure 3A) [130]. Interestingly, with normal expression of MyoD, the expression of myogenin is significantly attenuated at water–PFC interfaces in contrast to situations on elastic and/or viscoelastic environment. The findings emphasize the effect of fluid microenvironment on cellular differentiation. Furthermore, Jia et al. reported an adaptive protein (fibronectin) monolayer assembled at the liquid–liquid interface to emulate the dynamic adaptive native ECM (Figure 3B) [131]. The protein nanosheets can respond to cell traction forces with generation of the hierarchical fibre. hMSCs cultured on this interface show neuronal differentiation with increased phosphorylated focal adhesion kinase and nuclear localization of Yes-associated protein (YAP) compared with on the untreated liquid–liquid interface. The interfacial culture system provides new scenarios for the elucidation of the feedback mechanisms connecting ECM dynamic mechanics, biological signalling and long-term stem cell fate.

### 2.5 Topography

**Figure 4.**
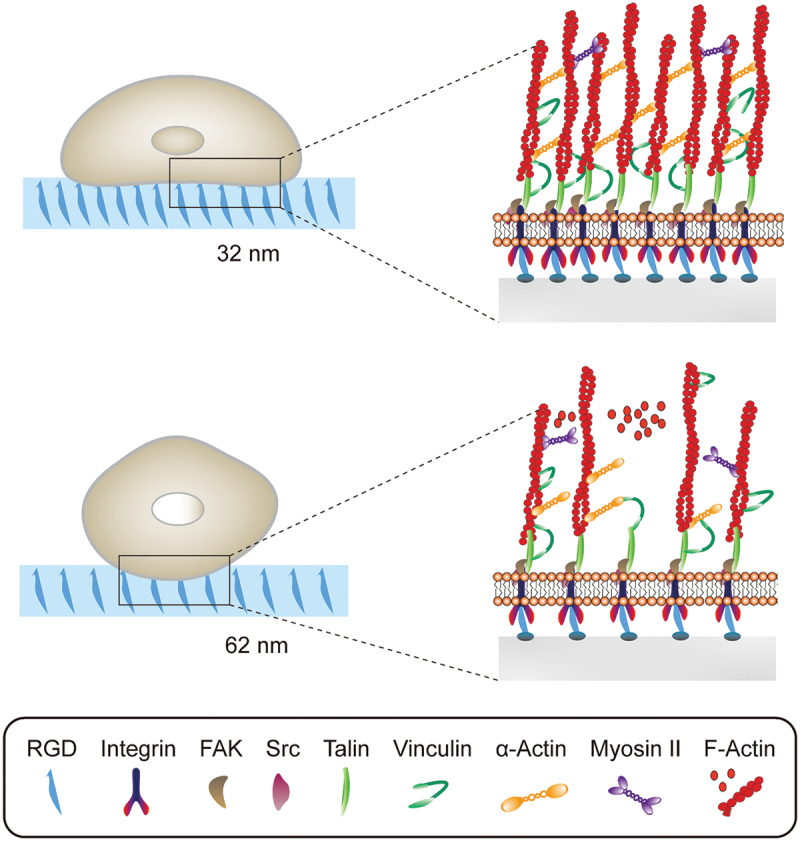
Different RGD nanospacings can alter hMSCs mechanosensing through differential coupling of FAK/Src/Rac1/myosin IIA/YAP/TAZ signalling pathways to support long-term changes in stem cell differentiation and state.

The topographical features are one of the biophysical cues that cells *in vivo* encounter ranging from protein folding to collage nanofibrils [132]. Understanding the cell–nanotopography interaction enables us to a better design of implant materials and stem cell therapeutics. For example, Dalby et al. fabricated five nanotopographies with varying degrees of disorder using electron beam lithography. Nanoscale disorder is found to promote MSCs osteogenic differentiation and production of bone minerals. In addition, the osteogenic differentiation profile of MSCs topographically stimulated and dexamethasone treated is distinct. Following this study, they hypothesized that absolute square lattice symmetry can retain MSC multipotency and phenotype. On this nanostructured surface, MSCs show minimum metabolism and repress differentiation-related canonical signalling, which may be regulated by extracellular signal-regulated kinase (ERK) and small RNAs. It can maintain the self-renewal and differentiation capacity of MSCs over 8 weeks. As shown in Figure 4, Han et al. investigate how hMSC fates are temporally modulated by different lateral nanospacings (from 30 to 60 nm) [133]. Smaller spacings recruit more activated focal adhesion kinase (FAK) and Src proteins, along with more myosin IIA. It promotes vinculin recruitment to more mature and stable focal adhesions and transduction of higher tension forces (by >5 pN/FA). Consequently, nuclear YAP/TAZ localization is increased with further increasing RUNX2 and decreasing β-catenin. They explain the underlying molecular mechanisms by which smaller nanoscale spacings bias the osteogenic commitment of hMSCs. Werner et al. constructed 3D macrotopographic cell culture chips to investigate the impact of surface curvature on hMSCs behaviours. hMSCs show distinct migration regimes on convex and concave structures with the latter obviously faster. It can be interpreted by different cytoskeletal forces. In addition, cytoskeletal forces of convex surfaces lead to substantial nuclear deformation and increasing lamin-A levels, which promote osteogenic differentiation on convex spherical structures.

**Figure 5.**
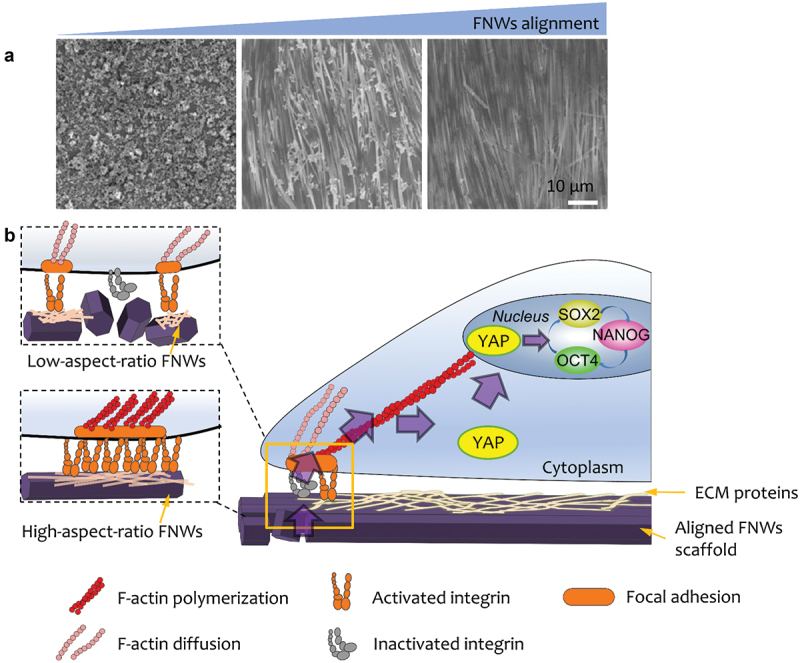
(A) SEM images of low-, medium-, and high- aligned FNWs nanopatterns. (B) Highly aligned fullerene nanowhisker nanopatterned scaffolds permit formation of mature focal adhesions and nucleus YAP translocation to promote hMSC multipotency retention.

In addition, topographical cues provide a unique strategy for the manipulation of stem cells without applying complex soluble factors or cellular reprogramming. Nanostructured substrates which lead to the retention of multipotency have been developed. As shown in Figure 5, Song et al. make use of fullerene nanowhiskers (FNWs) scaffolds with tuneable degree of alignment to culture hMSCs in large scale [134]. The LB assembly strategy is used for the fabrication of large-area nanostructured surfaces for the manipulation of stem cells. They demonstrate that the highly aligned FNWs prolong retention of hMSC multipotency in the absence of complex soluble factors or cellular reprogramming. Kong et al. cultured human adipose-derived stem cells (hADSCs) on a ZnO nanorod (ZnO NR) to maintain their stemness up to 3 weeks [135]. The underlying mechanisms can be explained by releasing Zn^2+^ from the ZnO NRs and stimulated KLF4, a Zn^2+^-binding gene, which lead to up-regulated NANOG and OCT4. Simulating the niche inside the blastocele, Hang et al. designed an anisotropic silk protein nanofiber-based hydrogel, which is capable of maintain mESCs’ pluripotency in the absence of LIF and MEFs [136]. Furthermore, the biomaterials establish a sustainable stemness-maintaining microenvironment through stimulating the secretion of autocrine cytokines. Their findings emphasize nanotopographical cues as regulators of stem cell fate.

### 2.6 Cell geometry

Because cell size and shape can affect cell physiology, cell geometry is established as a biophysical regulator of cell behaviours and fate. Stevens ’s group demonstrates that cell geometry contributes to variety in cytoskeleton networks and thus regulates plasma membrane lipid raft [137]. These biophysical changes trigger activation of Akt/PKB signalling and further regulate cell-geometry-dependent MSC differentiation. Their findings clarify the relationship between cell geometry and stem cell behaviour. Using polymer pen lithography, Cabezas et al. designed nanoscale anisotropic patterned matrix to direct the arranged formation of focal adhesion, which in turn facilitates the most organized cytoskeleton [138]. It is demonstrated that anisotropic focal adhesions increase MSC contractility and direct stem cells different lineage commitment.

### 2.7 Chemical properties

Except the biophysical cues above, the small-molecule functional moieties tethered on hydrogels are verified to alter hMSC morphology, protein and gene expression [139]. It can also control hMSC bidirectional lineage commitment. Tanaka’s group recently developed a double-network hydrogel composed of poly(2-acrylamido-2-methylpropanesulfonic acid) (PAMPS) and poly(*N,N’*-dimethylacrylamide) (PDMAAm) [140]. It can primely mimic niche of cancer stem cells (CSCs) with both soft and tough features and rapidly reprogram six human cancer cell lines into CSCs within 24h of seeding on the gel. More than that, the molecular machinery of cancer cell reprogramming, which clarified through double-network hydrogels, provides potential targets for reagents that specifically eradicate CSCs, such as platelet-derived growth factor receptor and osteopontin.

## 3 Biomaterials for immunoregulation

### 3.1 Bioactive materials for immunoregulation

Tissue repair and regenerative medicine are largely focused on stem cells. But immune cells play an equally important role in tissue repair. In early historical stage of tissue repair, immunological rejection is generally concerned for foreign grafts and transplanted cells or tissues. Immune response is considered as an obstruction that needs to be overcome. However, recent studies show that the regulation of immune responses by dampening pro-inflammatory or tissue-damaging responses and by activating anti-inflammatory or tissue-regenerative responses can greatly improve the tissue repair process [141].

At the tissue damage site, neutrophils and macrophages are early responsive immune cells. Neutrophils clean up damaged cells and ECM by enzymes and remove cell debris by phagocytosis. Macrophages have multiple phenotypes from pro-inflammatory M1 to anti-inflammatory M2 [142]. After tissue damages, pro-inflammatory cues such as interferon-γ (IFN-γ) and tumour necrosis factor (TNF) can activate M1 macrophages [143]. They can take part in phagocytosis and angiogenesis. However, a prolonged presence of M1 macrophages triggers chronic inflammation and delays tissue repair. M2 macrophages can be activated by cytokines such as interleukin-4 (IL-4), IL-10, IL-13 or IL-33 [144]. They can stabilize angiogenesis and stimulate ECM assembly and remodelling, and thus promote the tissue repair process. However, a prolonged presence of M2 macrophages can result in excessive fibrosis.

**Figure 6.**
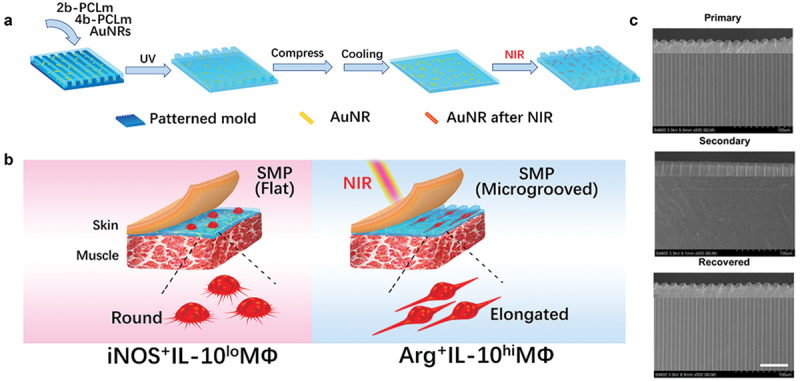
Study of the effect of Au nanorods (AuNRs)/polycaprolactone (PCL) thin films on macrophage phenotype. (A) Preparation and dynamic topography of shape memory AuNRs/PCL thin films. (B) Effects of topography of SMP on phenotypic changes of macrophages in rats. NIR triggered the transformation of SMP surface from flat to micro groove, which promoted the macrophage phenotype from iNOS^+^iL-10^lo^ MΦ (circular) to Arg^+^iL-10^hi^ MΦ (elongated) polarization. (C) SEM images of dynamic topography of SMP: primary micro-groove film, secondary flat film after compression and cooling, and NIR-triggered recovered micro-groove film. Scale bars are 50 μm [148]. Copyright 2019 American Chemical Society.

Besides the cytokines, macrophage polarization can be directed by biophysical cues such as stiffness, alignment and topography. Chen et al. cultured bone marrow-derived macrophages on PAAm gels with varying stiffness. They found that macrophages showed pro-inflammatory M1 on collagen fibres stiffness-like soft substrates (2.55 ± 0.32 kPa) and anti-inflammatory M2 on osteoid stiffness-like rigid substrates (63.53 ± 5.65 kPa) both *in vitro* and *in vivo*. Low-stiffness substrates stimulate macrophages to produce more reactive oxygen species (ROS), promote the activation of NF-κB signalling pathway and contribute to the polarization of the M1 phenotype. Snedeker’s group found that disordered polycaprolactone nanofibers alone direct macrophage polarization to pro-inflammatory M1, which can be enhanced by dynamic mechanical loading [145]. Randomly oriented fibre substrates can enhance tendon fibroblast response to paracrine signals of M1 macrophages, which is manifested in the up-regulation of matrix metalloproteinase (MMP) gene expression [146]. The highly aligned electrospun nanofiber tended to down-regulate the expression of MMPs. Their study indicated that macrophages as mechanosensory cells regulate tendon repair process. By high-throughput screening of 2,160 surface topologies, Alexander’s group found that micropillars 5–10 µm in diameter were more conducive to macrophage attachment [147]. The pillar size and the micropillar density affect the polarization of macrophages and thus can be used to regulate macrophages from pro-inflammatory to anti-inflammatory phenotypes. The balance of macrophage M1 and M2 is important for tissue repair. Smart materials with healing-match regulation of macrophage phenotype are beneficial for the tissue repair. As shown in Figure 6, Gao’s group developed shape-memory polycaprolactone/Au nanorods film whose surface can dynamically change, shifting from a flat surface to a microgroove under near-infrared irradiation [148]. The dynamic change of material surface induced the elongation and phenotype change of macrophages. This consequently upregulated expressions of arginase-1 and IL-10, achieving optimized tissue healing effect. Therefore, biophysical cues including stiffness, alignment and topography provide a new perspective for the design of ‘immune-instructive’ biomaterials for implantable medical devices and tissue repair.

Bioactive materials can be used as artificial niches for recruitment, homing and modulation of local host cells. Like drug delivery, synthesized scaffolds can carry soluble factors to modulate local microenvironments of disease sites. For example, anti-inflammatory cytokines IL-4 and IL-10 conjugated gold nanoparticles are used to treat muscular dystrophy [149]. Direct injection of IL-4 or IL-10 is suffered from unfavourable pharmacokinetic profiles. This needs repeated infusions accompanied by systemic side effects. After injection, IL-4/IL-10 conjugated gold nanoparticles are allowed to be localized distribution throughout the target muscle. Especially IL-4 conjugated gold nanoparticles recruit more T cells, especially the CD4+/CD8− helper T cells. The increased presence of T_reg_ plays an important role in improving histology and strength of chronically injured muscle. Tao’s group developed a macroporous silk gel scaffold that released sitagliptin to promote bone regeneration in diabetics [150]. Sitagliptin induces the polarization of macrophages to M2, thus alleviating the injury of osteoblasts to titanium graft and promoting bone integration. Compared with oral agents, sitagliptin released by silk fibroin scaffolds modulates macrophage phenotypic transformation and helps reduce inflammation at the site of injury.

With no exogenous growth factors, inherited biophysical and biochemical properties of implanted materials are found to regulate biological activity of local host cells. This enables biomaterial-based therapeutic treatments. With highly concentrated negatively sulfated groups, heparin interacts with many proteins to regulate physiological and pathological processes including inflammation, anticoagulation, lipid metabolism, angiogenesis, and neural regeneration [151]. Liu’s group developed a heparin-like semisynthetic sulfated chitosan (SCS) to promote angiogenesis and treat ischemic disease [152]. SCS is a highly bioactive biomaterial, which can mediate macrophage polarization to M2 through the STAT6 signalling pathway. SCS activates the vascular endothelial growth factor (VEGF) – VEGF receptor 2 signalling pathway of macrophages, making macrophages secrete endogenous VEGF to induce angiogenesis. Bioactive glasses, composed of SiO_2_, CaO, Na_2_O, and P_2_O_5_, have widely used in both hard and soft tissue repair, such as bone and skin regeneration [153]. Li’s group prepared a sodium alginate hydrogel containing bioactive glass (BG/SA) that induced macrophages to polarize into the M2 phenotype in vitro and in vivo, enhancing the secretion of regenerative cytokines and chemokines [154]. BG/SA hydrogel can further recruit fibroblasts and endothelial cells by regulating macrophages and promote angiogenesis and extracellular matrix remodelling, contributing to wound healing.

**Figure 7.**
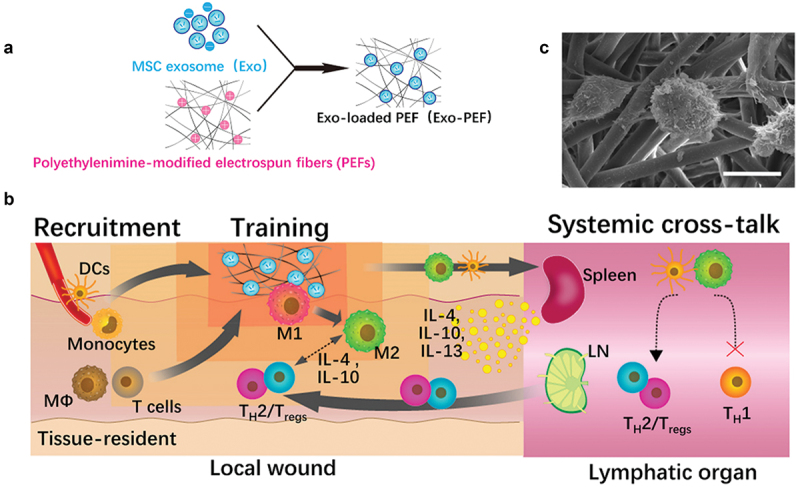
Preparation of EXO-PEF scaffold and its potential mechanism. (A) MSC exosomes were immobilized with PEI-modified electrospun fibres (PEFs) scaffolds by electrostatic interaction. (B) Potential immunomodulatory mechanism of EXO-PEF scaffold promoting tissue repair and regeneration: (i) recruitment of immune cells, (ii) training and promoting phenotypic transformation of immune cells, (iii) secretion of anti-inflammatory factors, and (iv) regulation of distant immune organs. (C) EXO-PEF cultured bone marrow-derived macrophages in vitro with lipopolysaccharides (LPS) simulation [156]. Copyright 2021 AAAS.

As an alternative to live cells, MSC-derived exosomes exhibit the immunomodulation function, involving promoting the M2 phenotype, T_reg_ population, and T_H_2 immune responses [155]. By loading MSC-derived exosomes on the fibrous polyester porous structures, Luo’s group constructed a multifunctional scaffold that stores exosomes and transmits signals (Figure 7) [156]. The scaffold plays a role in recruiting immune cells to the wound surface, and exosomes regulate the phenotype of immune cells. Therefore, the multifunctional scaffolds synergistically promote the M2 phenotype polarization of macrophages and the activation of regulatory T cell responses, which is beneficial to tissue repair.

**Figure 8.**
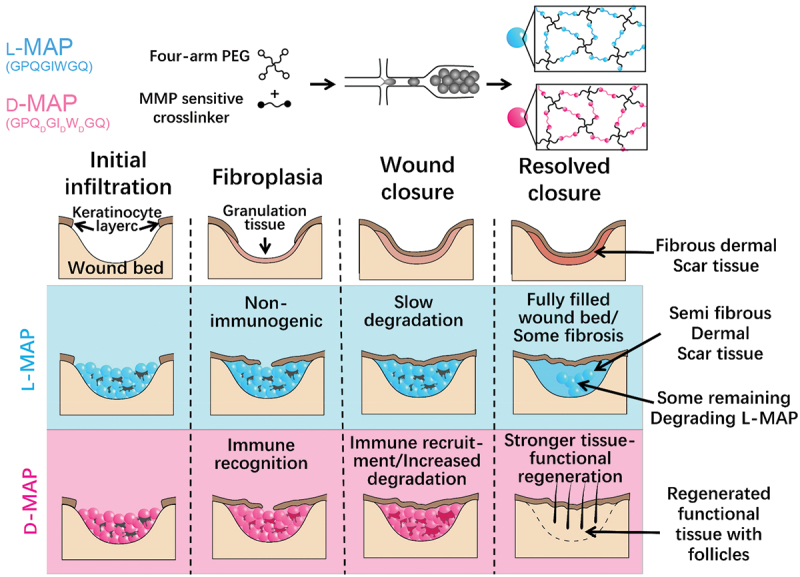
Schematic of MAP hydrogel preparation process and its effect on wound repair. (A) Diagrams of the preparation process of hydrogel microspheres. (B) Effect of L-MAP or D-MAP hydrogel applied to wound-healing model. Compared with L-MAP, D-MAP hydrogel can recruit immune cells and activate adaptive immunity, which further promotes tissue remodelling and regeneration and accelerates the in vivo degradation of D-MAP.

Although modulation of innate immunity like macrophage phenotype by bioactive materials is reported in many studies, the adaptive immune system plays an important role in tissue repair and regeneration. Using microfluidic methods, Griffin et al. developed an injectable annealed microporous interconnected gel scaffold [157]. It can be used for 3D cell culture *in vitro* with the ability of promoting cell proliferation. When injected into the body, it can promote wound healing and tissue regeneration. To slow down the degradation of microporous annealed particle (MAP) in vivo, they changed the chirality of the crosslinking peptides from L- to D-amino acids (D-MAP) (Figure 8A) [158]. Surprisingly, they found that D-MAP degraded faster in vivo but produced a better tissue regeneration effect. The reason is that D-MAP induces adaptive immune response in vivo, leading to wound healing with tissue functional recovery (Figure 8B). This indicates the important role of adaptive immunity in biomaterial-mediated tissue regeneration.

### 3.2 Biomaterials regulate immunomodulatory functions of MSCs

**Figure 9.**
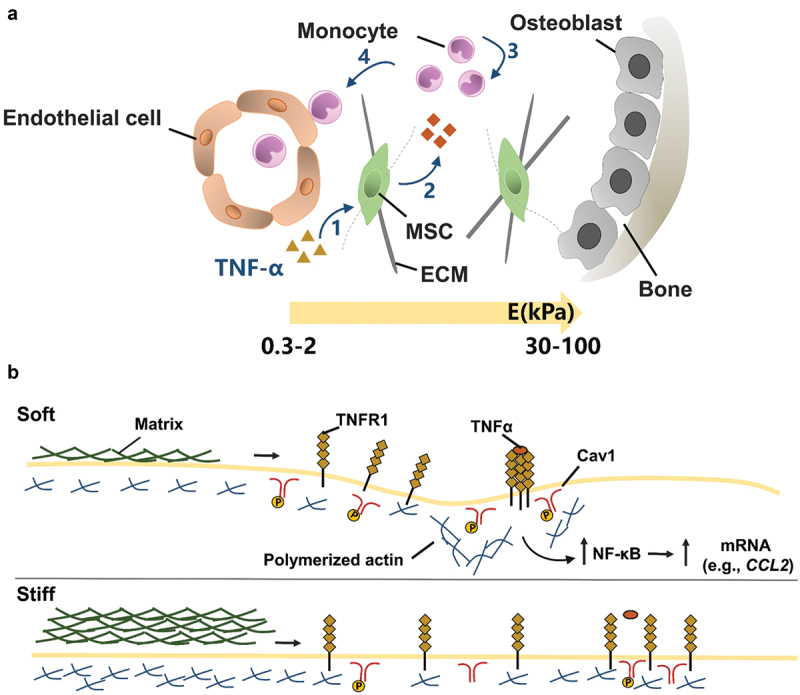
The effect of matrix stiffness on the immunomodulation of MSC. (A) Schematic of the effect of matrix stiffness on the immunomodulation of MSC. Under inflammatory conditions, ECM stiffness affects (1) the activation of MSC by inflammatory factor TNFα, and stimulates MSC to produce paracrine factors, thus affecting (3) the recruitment and (4) transport of monocytes. In bone marrow, the ECM near the endothelial cell region is softer (Young’s modulus E = 0.3–2 kPa), while the ECM near osteoblast region is stiffer (E = 30–100 kPa). (B) Mechanism of matrix stiffness regulating TNFα binding to cell surface. The soft matrix mediates the redistribution of actin polymerizations, and TNFα stimulates lipid rafts to promote TNFR 1 aggregation, thereby enhancing downstream gene expression. However, on stiff matrix, actin polymerization was reduced, hindering the binding of TNFα to cell surface TNFR 1.

Besides multipotent differentiation, immunomodulation is an important biological function of MSCs. MSCs can modulate immune cells to treat multiple immune- and inflammation-mediated diseases [159]. MSCs from the bone marrow or fat are expanded in vitro and then are administered to ameliorate diverse inflammatory pathologies [160]. Recent studies are increasingly realized that biophysical signals from the extracellular matrix can regulate the immunomodulatory functions of MSCs. As shown in Figure 9, Wong et al. found that the stiffness of alginate-RGD hydrogel regulated inflammatory activation of MSCs to induce monocyte production and trafficking [161]. In the presence of TNF, soft matrix can promote TNF receptor aggregation, and thus increase NF-κB activation and MSC paracrine. The enhancement of the paracrine effect promotes the recruitment and chemotaxis of monocytes. Soft polyacrylamide hydrogels (~0.5 kPa) promoted the secretion of immunosuppressive factors and nutritional factors of MSCs compared with rigid hydrogels (~200 kPa) [162]. Furthermore, soft matrix induces macrophage M2 polarization and human umbilical vein endothelial cell tube formation and chemotactic migration, thus promoting tissue regeneration.

Besides the elasticity, fibrous topography of scaffolds is another key biophysical signalling that can regulate the immunomodulatory functions of MSCs. Su et al. investigated the effect of alignment characteristics of electrospinning fibres on rat adipose-derived MSCs to produce pro-angiogenic and anti-inflammatory paracrine factors [163]. They collected conditioned-medium from random, aligned and mesh-like electrospinning fibres, respectively. The conditioned-medium was applied to the cultures of endothelial cells and macrophages *in vitro* and a skin wound-healing model *in vivo*. Among them, the conditioned-medium from MSCs cultured on mesh-like fibres has the most obvious effect on the anti-inflammatory response of macrophages, while MSCs cultured on aligned fibres have a stronger ability to promote angiogenesis. Aligned fibres activate the FAK-ERK1/2 and YAP/TAZ signalling pathways of MSCs, which in turn mediate immunoregulation of MSCs by aligned fibres [164]. Taken together, soft matrix and aligned fibres promote the secretion of anti-inflammatory and pro-angiogenic factors of MSCs, thus promoting tissue repair.

Cell microenvironments including the extracellular matrix, neighbouring cells and soluble cues, collectively affect regenerative potential of MSCs. Geissler et al. compare paracrine effects of MSCs in nanoporous hydrogels and microporous hydrogels [165]. They found significantly upregulated paracrine effects of MSC in microporous hydrogels in the presence of growth factors IGF. This is due to the cell–cell interaction of MSCs in microporous hydrogels. By conjugating N-cadherin mimicking peptides to nanoporous hydrogels, MSCs’ paracrine effects can be improved. These results indicate the collective effect of the matrix, neighbouring cells, and soluble factors on the regenerative potential of MSCs.

**Figure 10.**
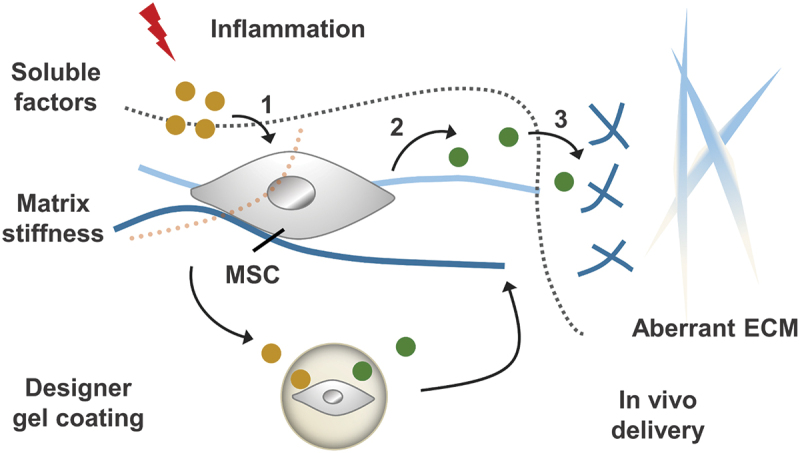
Inflammatory stimulation alters chemical and physio-mechanical signals in the cellular microenvironment, which influences MSC behaviours including: (1) the process of receiving soluble factors and (2) the paracrine effect of MSC. By designing gel coatings with defined chemomechanical cues, MSC regulation can be improved to (3) correct aberrantly ECM remodelling.

Clinical studies of MSCs transplanted systemically have limited success. One of the big questions is that most MSCs transplanted systemically are trapped in the microvasculature of filter organs (such as the lungs) and the short residence time of infused cells [166]. Encapsulation of single cells in a thin hydrogel layer can increase the residence time of MSCs in vivo but not affect cell viability and the diffusion of soluble factors [167]. By increasing MSC density in microgel, the residence time of MSC in vivo can be prolonged significantly, and the multicellular microgel can affect expression of hematopoietic and immunomodulatory genes [168]. In the presence of TNF-α, MSCs coated with a soft conformal gel activate the JNK signalling pathway (Figure 10) [169]. This pathway is associated with the paracrine of soluble interstitial collagenases, such as matrix metalloproteinases 13 (MMP13), which contributes to the degradation of fibrotic matrix and matrix repair. In the presence of donor MMP13 and host TNF-α, treatment of MSC with microgel wrapping can effectively inhibit fibrosis lung injury in mice model. Together, gel encapsulation of cells with predefined chemo-mechanical cues can program cellular functions for desired therapeutic outcomes.

## 4 Biomaterials direct the self-organization of pluripotent stem cells for organoid

Pluripotent stem cells, such as human ESCs and iPSCs, are important for regenerative medicine and disease models. Human organoids generated from pluripotent stem cells provide unique strategies to capture important features of tissues in vivo [170]. To generate human organoids, pluripotent stem cells are cultured and differentiated in Matrigel-based substrates. Secreted by Engelbreth–Holm–Swarm mouse sarcoma cells, Matrigel is a complex mixture of various ECM proteins, proteoglycans and growth factors [171]. This tumour-derived Matrigel with un-uniform composition and structure has limited clinical translational potential. The mechanism that the biophysical cues of Matrigel affect the growth and differentiation of pluripotent stem cells remains unclear.

Cruz-Acuña et al. developed a synthetic hydrogel, a four-armed maleimide-terminated PEG hydrogel (PEG-4MAL) [172]. The hydrogels support human intestinal organoid generation from human embryonic stem cell- and hiPSC-derived spheroids in vitro without embedding in Matrigel and support their viability, expansion and development. The mechanical properties and adhesive ligand type of the hydrogel are important factors that affect human intestinal organoids viability, expansion and development. In addition, PEG-4MAL can serve as an injectable delivery vehicle to deliver human intestinal organoids using a colonoscope, resulting in improved colonic wound repair in vivo.

Lutolf’s group established tubular hydrogel scaffolds composed of a mixture of type-I collagen and Matrigel [173]. Collage I provides a stiff, adhesive substrate, and Matrigel contains the key constituents of the native basement membrane. These hydrogels are integrated in a perfusable hybrid microchip system that consists of an elastomeric device. It can be colonized with intestinal stem cells to form tube-shaped functional intestinal epithelium with a spatial arrangement of crypt- and villus-like domains similar to that in vivo. Moreover, the self-organized epithelium forms a perfusable lumen. This prolongs tissue lifespan and allows modelling host–microorganism interactions.

**Figure 11.**
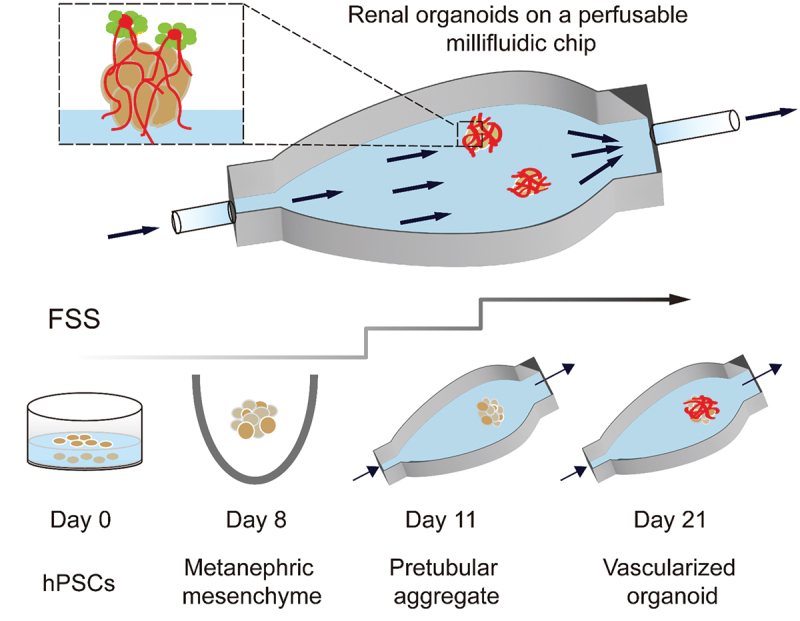
Developing renal organoids cultured within a perfusable millifluidic chip and subjected to controlled FSS exhibit enhanced vascularization and maturation.

As shown in Figure 11, Homan et al. used 3D-printed millifluidic chips to house the developing kidney organoids embedded in engineered ECM [174]. Under high fluid flow, kidney organoids showed enhanced vascularization and increased mature perfusable lumens, podocyte and tubular compartment morphogenesis compared with that in static controls. Fluid flow inducing vascularization and morphological maturation of kidney organoids in vitro provides a new dimension for studies of kidney development, disease, and regeneration.

Matrix stiffness and viscoelasticity are revealed as potent regulating factors for stem cell morphogenesis. Sorrentino et al. establish a PEG-based 3D hydrogel system mimicking hepatic niche [175]. The hydrogels support efficient derivation of both mouse and human liver organoids, even without any animal-derived matrices. Notably, matrix stiffness directing liver organoid growth requires YAP activated by SFKs instead of actomyosin contractility contrary to that in intestinal organoids. In addition, they tuned PEG hydrogels to model fibrotic liver mechanics, providing evidence on how aberrant mechanical cues impacting liver. Chaudhuri et al. use viscoelastic alginate hydrogels with independently tuneable stress relaxation, stiffness and RGD ligand density to study how these properties control hiPSC morphogenesis in 3D culture [176]. They demonstrate that higher RGD density and fast stress relaxation promote hiPSC apicobasal polarization and apicobasal polarization, while stiffness has no influence on hiPSC morphogenesis. Notably, hiPSCs can maintain pluripotency in the hydrogels until day 14 of culture, much longer than reported in reconstituted basement membrane matrices. These findings provide new insights into how we can take advantage of the biophysical cues of engineered biomaterials to construct organoids.

## 5 Summary and perspectives

As described above, this review is divided into four parts. General backgrounds for the preparation of advanced materials with nanoarchitectonics are described in the introductory part where importance of materials impacts to stem cells is emphasized as a guidance for future bio-hybrid nanoarchitectonics. In the following sections with concrete descriptions for materials-based control of stem cells, we firstly discussed the biomaterials for regulation of stem cell fate. Besides multipotent differentiation, immunomodulation is an important biological function of MSCs. MSCs can modulate immune cells to treat multiple immune- and inflammation-mediated diseases. We then summarized the recent advances of the regulation of the immunomodulatory functions of MSCs by biophysical signals. In the third part, we discussed how biomaterials direct the self-organization of pluripotent stem cells for organoid. Tissue repair and regenerative medicine are largely focused on stem cells. Besides biochemical factors, biophysical cues are increasingly being recognized as new dimensions to regulate stem cell behaviours and fate. In this review, we summarized how the biophysical properties of biomaterials regulate stem cell fate and function, including stem cell differentiation, stemness maintenance, immunoregulative function of MSCs, and stem cell morphogenesis for organoid. Bioactive materials are constructed which mimic the biophysical of *in vivo* microenvironment such as elasticity, viscoelasticity, biodegradation, fluidity, topography, cell geometry, etc. Stem cells interpret these biophysical cues by different cytoskeletal forces. Different cytoskeletal forces lead to substantial transcription and protein expression, which affect stem cell fate and function.

These facts can be interpreted from different viewpoints. The studies described above indicate that stem cells are sensitive to perceive natures of contacted materials. Therefore, stem cells can be active components for materials nanoarchitectonics with delicate regulations of interactive information exchanges between materials and cells. Transmission of signals and information from material surface to living cells can induce complicated events of signal transductions and material conversions within the cells, which potentially produces advanced functional outputs. Regulations of stem cells could not be utilized only for tissue repair and regenerative medicine but also potentially for production of advanced materials systems. Materials nanoarchitectonics with integration of stem cells and related biological substances would have high impacts in science and technology of advanced materials.
